# Does organizational culture influence employee productivity at the local level? A test of Denison's culture model in Ghana’s local government sector

**DOI:** 10.1186/s43093-022-00145-5

**Published:** 2022-09-06

**Authors:** Juliana Abagsonema Abane, Ronald Adamtey, Virceta Owusu Ayim

**Affiliations:** 1 Department of History and Political Studies, Kwame Nkrumah University of Science and Technology-KNUST, Kumasi, Ghana; 2 Department of Planning, KNUST, Kumasi, Ghana; 3 Sekondi-Takoradi Metropolitan Assembly (STMA), Sekondi, Western Region Ghana

**Keywords:** Organizational culture, Employee productivity, Local authorities, Ghana, Theory X and Y

## Abstract

There is mixed evidence that low levels of productivity at the local government level are not common with organizations with strong cultures as these are less prone to any externalities. The paper investigated the link between organizational culture and employee productivity from the perspectives of employees of the Sekondi-Takoradi Metropolitan Assembly (STMA) in Ghana. The study used the quantitative approach with descriptive and cross-sectional designs. The simple random and stratified sampling techniques were used to select 132 respondents from the STMA using a self-administered survey questionnaire between August 2020 and December 2020. Denison’s 1984 model of organizational culture was adopted to measure organizational culture while employee productivity was measured by target achievement, available resources and hours of work in a week. With the support of the “SPSS version 22,” the study used descriptive statistics, bivariate analysis and ANOVA tests with hypotheses using standard regression methods. The findings indicate a strong and positive culture of mission, involvement, adaptability and consistency in the STMA. Further, two hypotheses of the study were supported while one hypothesis failed to reject the null hypothesis. However, the relationship between the culture of adaptability and employee productivity was negative but statistically significant; hence, the research hypothesis on this variable was modified to reject the null. Denison’s culture model, which was used to measure organizational culture, was less effective in explaining the variance in the dependent variable as a combined variable.

## Introduction

Local governments in many countries in sub-Saharan Africa have issues with employee productivity in the past five decades. In recent times, employee productivity has suffered because of the COVID-19 pandemic [21, 22]. Ways to enhance employee productivity appear to be eluding governments, public policy and public administration experts in developing countries. This is largely so as current literature on employee productivity and popular models such as Denison’s Culture Model do not provide sufficient understanding. Key argument by the Denison’s Culture Model is that organizational culture is sufficient to understanding employee productivity. But does this model work in all contexts and does it adequately explain employee productivity at the local level in Ghana?

Ghana has issues with low-productivity problem in the public sector, and there has been increased call to enhance employee productivity in the public sector to improve service quality [46]. As is the case in many developing countries, the public sector in Ghana is the largest employer providing social services with tax revenue that the private sector does not, yet it is unable to mobilize sufficient tax revenue due to low employee productivity in the tax sector. According to the government’s budget declaration, tax revenue has been falling, and the government has failed to fulfill its revenue targets in 2017, 2018 and 2019 [40]. Such poor performance of public sector workers in developing countries such as Ghana has been attributed to the unavailability of materials and logistics, poor employee remuneration and motivation, political interferences and weak technical and technological capacity [7, 14, 46].

Although these factors may explain the phenomenon, it appears that culture plays a critical role [20], yet this does not appear to be adequately explored to understand employee productivity in the Ghanaian public sector. In most societies, both advanced and developing countries, people are surrounded by a culture which shapes their attitudes to work and explain how productive they can be. As a result, organizations operate as a social unit with their own culture that shapes employee attitudes. Consequently, this research uses [17] organizational culture framework, which focuses on involvement, consistency, adaptability, and mission and their ability to boost employee performance [10].

Organizational culture, also referred by many as corporate or company culture, is explained to be a set of qualities that distinguishes one institution from others by establishing its distinctiveness. Torres [48] sees it as a set of common values and standards that employees and groups in an organization adhere to and express in their interactions. There is the consensus that organizational culture is a tool for regulating employee conduct [11, 40].

Employees with a positive organizational culture have comparable attitudes, ethical values and beliefs, whereas employees with a negative corporate culture have different ethical values and beliefs [49]. Also, companies may succeed if their company culture and productivity management systems are in sync. Although organizational culture is not new concept, there is evidence to suggest that local authorities are unable to understand and appreciate how a strong organizational culture will impact their productivity. Consequently, they do not make the efforts to build a strong culture that will improve their productivity levels. This is undermining the country’s decentralization policy and local governance reforms implemented in the past four decades to deliver as envisaged by the reforms. Consequently, this study is aimed at exploring the evidence on the significance of culture and its impact on local level employee productivity. The main contributions of this study are that it helps to affirm and deepen our understanding of Denison’s model on how organizational culture matters in advancing the frontiers of employee productivity enhancement discourse. The objective of this study is to use the Sekondi-Takoradi Metropolitan Assembly (STMA) as a case to answer the question:

R1: How does organizational culture impact employee productivity in the context of a local government organization like the STMA?

The paper is organized into five sections: introduction, literature review, methods, results and discussions as well as the conclusion.

## Theoretical review

In this section, the literature is examined focusing on the theoretical, conceptual and empirical investigations. In addition, the notions of organizational culture and employee productivity are examined first together with McGregor’s theories X and Y and Denison's organizational cultural model. Empirical studies of factors influencing employee productivity as well as the impact of organizational culture on employee productivity are also discussed (see Tables 1 and 2). These two theories are key to this study because employee productivity is explained by McGregor’s X and Y through the content hypothesis which helps to explain the unique aspects that inspire and motivate a person's productivity-enhancing activity. Similarly, Denison's organizational model aids in the comprehension of organizational culture.

**Table 1:** Employee productivity Scale

Variable	Item description	Mean	SD
Employee productivity (EP)		3.88	3.037
	EP1. I am able to meet my annual job targets	3.030	1.487
	EP2. I complete all my job tasks on time	2.742	1.390
	EP3. The organization gives me the financial resources needed	3.235	1.720
	EP4. I achieve less than the resources given me	3.257	1.189
	EP5. I am able to achieve more than the resources at my disposal		
	EP6. I work at least 40 h a week	2.970	1.097
	EP7. The organization gives me the material resources needed	2.871	1.250
		3.121	1.370

*N* = 132, Min = 1, Max = 5

**Table 2:** Organizational culture scale

Variable	Sub-indicator	Item	Mean	SD
*Organizational culture (ORGCLT)*				
	Consistency (CONY)		1.641	0.657
		CONY1. I share a set of attributes that creates a sense of identity with the institution	1.977	1.080
		CONY2. Assignments given to me are consistent with my strength and interest	1.613	0.737
		CONY3. The values in the institution are consistent with the organizational goals	1.333	0.738
	Involvement (INT)		2.141	0.279
		INT1. The institution empowers and engages me		
		INT2. The institution ensures I make input into decision-making	1.818	0.675
		INT3. We work as a team in this institution	2.325	0.624
	Adaptability (ADBY)		2.280	0.529
		ADBY1. I can work in this institution for a longer period	2.369	0.991
		ADBY2. I am able to learn from my mistakes	2.090	0.746
		ADBY3. I am able to cause changes in my roles to achieve the job task	2.485	1.511
	Mission (MION)		2.530	1.655
		MION1. I am aware of the mission of the institution	2.361	1.481
		MION2. I am aware of the institution’s strategic policy direction	2.136	1.517
		MION3. I am aware of the organization’s goals	2.379	1.449
			2.568	1.681

*N* = 132; Min = 1, Max = 5

### McGregor’s theory X and Y

Douglas McGregor makes two contradictory insights about human work behavior [28, 31]. According to McGregor, firms utilize one of two techniques to regulate employee behavior: theory X or theory Y. According to theory X, it is the responsibility of management to ensure that the use of money, materials and employees is organized in a way that meets the organizational purpose or goal. Also, employees have an inherent attitude not to perform at optimum if work environment and management weaknesses make it so [43]. Therefore, there is a need for management to direct and take steps to discourage the attitude of employees in avoiding work. Hence, high productivity can be achieved by controlling the actions and behavior of employees [38, 39]. In support of theory X, [28] found an inherent lazy attitude of workers which affected employee performance when no direction was given, arguing that such types of employees need to be rewarded, persuaded, coerced and directed before the organizational goal can be achieved.

McGregor’s theory Y focuses on how resources such as money, materials and employees are organized effectively to aid the achievement of corporate goals. In theory Y, employees are believed to be committed to organizational goals and objectives and are so willing to achieve them. In this light, the theory states that management must take advantage of this situation and provide employees with the right working conditions and resources needed to achieve the corporate goal (see Table 1). Thus, Theory Y assumes that management will support employees by giving them the right organizational values that help them develop their potential. According to Mullins [32], providing a stable and positive organizational culture is a requirement in ensuring increased productivity of employees.

### Denison’s organizational cultural model

According to Denison, Haaland and Goelzer [18], the four main principles that underpin organizational cultural theory are consistency, engagement, flexibility and mission. The model defines involvement or participation as the cultural attribute that aims to empower individuals, organize the company around groups or teams and improve human talents on all levels. For the participation indicator to be legitimate, managers, supervisors and employees must be committed to the business and its mission.

Denison and Mishra [19] note that organizations interact with the environment in a sociocultural framework, which has an impact on the lives of employees. And since people are bound together by their culture, organizational culture can be a potent tool for influencing employee worldviews, which has a direct impact on the organization where they work.

Furthermore, Denison [17] argues that the institution's culture must be consistent because of the underlying ideals, beliefs, principles and values. The stability of a company's culture either attracts or repels employees. People’s conduct is rooted in a set of underlying beliefs, and managers and employees are adept at finding consensus even when opposing viewpoints exist [15]. As historical accounts show, civilizations with consistent cultures and well-coordinated and integrated systems tend to be more successful and efficient [49]. Consistency has been used to explain the foundations of a productive corporate culture by many [15, 17, 49]. In addition, organizations need to align their values and belief systems with performance goals to increase productivity. To a large extent, goal attainment is influenced by organizational culture alignment with individual and organizational goals as well as the integration of systems and people to create synergy to execute operational and tactical activities [49]. In this way, the synergy created through cascading organizational culture will impact employee commitment and control of task performance leading to an increase in productivity.

There are, however, some limitations to Denison's culture model. For instance, in terms of an organization’s ability to allow people to adapt to its culture, the model does not indicate how this process is achieved. This is because people can be productive if their culture is strong and adaptive, and at the same time, they can also be counterproductive if their culture is negative and their adaptation is low [18]. Similarly, adaptive firms may be willing to take risks and learn from their failures, as well as have the skills and experience to implement change which explains their speedy adaptive nature [2]. Adaptable organizations update their systems regularly to increase their collective responsibility to achieve corporate goals.

The third cultural feature, according to Denison, Nieminen and Kotraba [16], is organizational mission. The mission attribute is concerned with how a feeling of purpose, vision and mission can help a community or organization thrive by directing how people should behave. Because organizations act as societies, the mission trait further describes the requirement for firms to have a mission in which strategic and policy orientations are used to accomplish the goals. As a result, successful businesses must have a clear sense of purpose and direction that outlines the company's strategic goals and objectives [18].

These two theories are the foundation of this study. Consequently, involvement, consistency, adaptability and mission are the core variables that are used to measure organizational culture in this study (see Table 2).

### Employee productivity

There are many different explanations for employee productivity and employee performance is commonly used interchangeably with the term. According to [25], employee productivity is the employee production in a corporation with the help of resources. This indicates that an individual capacity to be productive can be said to be the ratio of the available resources that the institution provides to the effort exacted to produce a given unit of goods and services (see Table 1). The resource can be monetary, training or other things required to complete an activity. In the view of [25], employee productivity measures employee's efficiency when provided the required resources. As a subsequent outcome, the term emphasizes an employee’s efficiency. The time it takes to complete a task has an impact on an employee's efficiency [13]. And according to [22], employee productivity is the production of products and services that an employee creates within a specified timeframe. This shows that employee productivity can also be quantified in terms of hours of work time [44]. According to Sauermann [44], definitions of employee productivity can also depend on the industry. For example, employee productivity can be easily quantified in connection to the number of goods produced and the resources or inputs used in the manufacturing industry. In the service sector, this can be measured in terms of service quality [47].

Each organization in any sector, however, is at liberty to apply either quantity or quality measures, or both.

In addition, according to Leblebici [29], technological improvement can influence staff productivity through innovation, skill enhancement and efficiency, all of which are important contributory aspects to profitability and outcomes. As a result, some input characteristics, as well as the efficiency with which production resources are used, have an impact on staff productivity. In this scenario, two employees may have similar productive technology, but one will be more productive than the other because he or she has more money. When compared to the marginal product of labor, an employee's output or outcome agrees with the average product of labor.

In light of prior definitions of employee productivity, this study defines it as an employee's capacity to fulfill goals set in the job description and employment agreement in a given period. Workers' productivity can be viewed by comparing the man hours by an employee in a week to the total hours of task performance, as well as the efficiency with which they met weekly targets or goals. This kind of measuring can be used to see whether an employee's production is on track. Furthermore, the efficiency metric aids in determining an employee's ability to complete a task given the resources required or available [30]. These productivity measurements help management improve operations by allowing them to assess and quantify employees' working abilities in terms of the time it takes to accomplish a task or achieve a goal. These metrics are intended to give a clear and precise foundation for contrasting real and projected outcomes.

### Organizational culture

Schein [45] relates that organizational culture comprises three interconnected levels. First, at the bottom are assumptions that reflect the beliefs of nature and reality. The second is values, which are common principles and ideals. Lastly, at the top is visible and concrete characteristics of institutional culture. The term culture is used to describe how individuals feel, think and act in a particular way. A collection of people develops and interprets the same ways of doing things [41]. As a result, the term “culture” is now used in companies to refer to the traits, way of life, knowledge, language and social habits of people who work in the same organization.

Different schools of thought have also used culture, in general, to describe an organizational culture in different ways [1]. Organizational culture, for example, is viewed as a common symbolic system that incorporates cumulative mental products and unconscious mental processes that underpin cultural expressions by the structuralist school [9, 12, 37]. Similarly, the cognitive school of thought considers organizational culture to be a set of taught principles for perceiving, believing, evaluating and behaving [9]. This implies that an organization’s culture is made up of what people know or think and that this knowledge or belief impacts conduct in a way that is acceptable to all employees [26]. The functionalist school, on the other hand, claims that organizations are systems with objectives, purposes and needs that are always in contact with their surroundings. In the view of [19], an organization with a distinct cultural framework from the rest of society is what constitutes organizational culture. Also, most organizations have cultures that tend to bind individuals together as a unit and they act in like manner because the success or failure of the organization is dependent on it [6, 8]. It is also seen as the cooperative values, beliefs and principles of institutional members. As a result, it is influenced by history, product, market, technology, strategy, management style and country culture [4, 50].

Organizations with strong cultures, according to [44], are difficult to copy and have a competitive edge over institutions with weaker cultures. Nongo and Ikyanyonl [45] discovered that corporate culture is an important corporate strategy for the growth of businesses around the world. Daft [15] rightly puts it that the right culture results in a competitive advantage for an institution. Therefore, weak culture results in lower productivity, leading to institutional failure [12, 38, 39]. Hence, culture is an influential tool for regulating employee behaviors rather than rules and regulations. For instance, rules and regulations may not help in solving client–organization problems when there is the need to improve upon the quality of customer service or when to solve customer challenges. Rather, institutions must create a favorable culture to aid employees to think and produce solutions toward improving customer–institution relations.

According to Wambugu [53], a basic assumption shared by employees and managers may benefit the institution. This assumption must be transformed into values such as classlessness and good relationships. The visible and concrete characteristics of such values are the “open door” policy and an office arrangement that includes open spaces. As a result, Torres [48] suggests that studying an organization’s physical environment, employee relations, business regulations, reward systems and other observable elements might help one comprehend its culture. However, most often, a critical look at these observable elements may not give the true representation of the organization, because the composition of the institutional culture exists under a person’s level of awareness.

### Empirical review

A study on organizational culture and employee productivity of six (6) zonal and 36 state units in Nigeria found that there is a strong relationship between organizational culture and employee productivity [3]. The total variance explained showed the analysis of variance (ANOVA) test was statistically significant and a strong positive connection between employee dedication and organizational culture, as well as a decent organizational culture.

Ahmed’s [3] study of the telecom industry in Pakistan revealed that there is a statistically significant association between organizational culture and employee productivity. The research used some Telecom franchisees in Bahawalpur and measured an organizations' productivity using the balanced scorecard. The findings showed that all aspects of organizational culture had a significant impact on several aspects of organizational productivity. Similarly, Ng’ang’a and Wesonga [36] discovered that organizational culture had a substantial impact on educational institution performance in Kenya. Return on equity, asset and profitability was used to evaluate the performance. The study discovered that culture is a crucial component of good organizational performance and that there is a strong link between culture and performance.

In Pakistani universities, Mujeeb, Masood and Ahmad, [33] discovered a link between organizational culture and performance management techniques. The exploratory research approach was used to obtain data from 140 employees via primary means such as questionnaires. Inferential statistical procedures such as regression and correlation were utilized to analyze the data, which included both male and female faculty members. Involvement as a component of corporate culture was found to be substantially connected with consistency and adaptability.

Similar research of organizational culture and performance management procedures in information technology organizations was undertaken in Romania [42]. The Chi-square test and bivariate analysis of primary data from 82 workers in the information technology business in Bucharest, Romania, revealed that organizational culture characteristics had a moderate effect on performance management. Also, involvement, consistency, adaptability and mission were significant and positive on performance management, Consistency also has the greatest influence on performance management approaches.

In Ghana, the effect of organizational culture on performance provides substantial evidence [14]. The study used questionnaires and convenience and purposive sampling techniques to sample 185 nonrandom respondents from Ghana's capital. According to a Pearson correlation analysis, there is a strong positive association between corporate strategy and organizational culture and performance. Similarly, [34] used 60 employees from several firms to investigate the impact of company culture on employee performance. Employee performance and organizational culture were found to have a favorable and strong significant association, according to the t-tests and Pearson correlation matrices. The study also discovered that there was no statistically significant difference in employee responses to organizational culture and performance based on gender.

In addition, [1] investigated how organizational culture affects employee productivity in Nigeria using a descriptive mixed-methods research design, the ordinary least square (OLS) simple regression method showed that consistency was a significant determinant of an employees’ effectiveness. The mission of an institution also had a significant effect on job efficiency. The research also discovered a link between company culture and employee productivity.

**Fig. 1:**
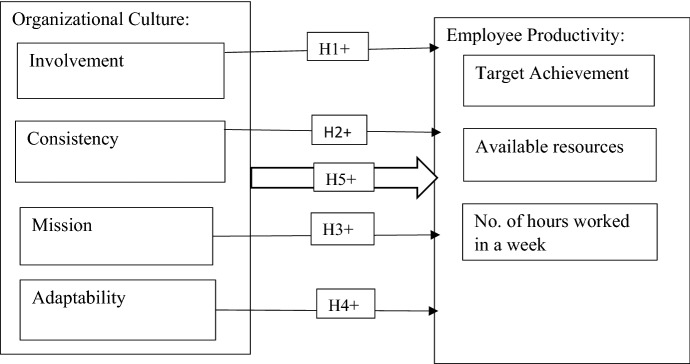
Research framework

Findings from the theoretical, conceptual and empirical reviews of the two variables are in tune with recent findings from empirical studies such as [5, 24, 52] which reveal that organizational cultural dimensions as put forward by Dennison’s 1984 have an impact on employee productivity. From the review of the variables, the following hypothesis and the research framework in Fig. 1 guided the study in the empirical data collection:

#### H_1_

There is a positive relationship between involvement and employee productivity

#### H_2_

The culture of consistency has a strong effect on employee productivity

#### H_3_

Increase culture of adaptability is positively related to employee productivity

#### H_4_

The mission of an organization has a positive influence on employees’ productivity

#### H_5_

There is a statistically significant relationship between organizational culture and employee productivity.

## Methods/experimental

### Research design

The study employed a quantitative approach with descriptive survey as the main tool. According to [35], the descriptive study approach helps to understand the features of a phenomenon that exists. A cross-sectional survey was employed for field data collection from the staff of the Sekondi-Takoradi Metropolitan Assembly (STMA).

### Sampling strategy

The study involved a target population of 636 staff of the STMA comprising staff in the following departments: Finance, Budget, Revenue, Works, Social Welfare, Waste/Environmental, Planning and Central Administration.

Using these departments, the study grouped the staff into Finance/Budget/Revenue, Works/Social Department, Waste/Environmental/Planning and Central Administration. Following this, a simple random procedure was used to select respondents from each stratum who made up the sample size. This method of sampling was adopted to guarantee that each employee had a chance of being chosen. It also allowed for the representativeness of the sample to ensure reliability and validity of the data. A sample size distribution table involving a sample population of 636 using an error term of 5% [26] produced a sample size of 234 to which questionnaires were self-administered between August 2020 and December 2020.

In all, 132 questionnaires were retrieved for the analysis representing a 56.41 percent response rate. Table 3 shows the sample size distribution.

**Table 3:** Summary of Study Sample

Departments	Population (N)	Sample (*n*)
Finance/budget/revenue	34	13
Works/social department	109	40
Waste/environmental/planning	178	65
Central administration	315	116
Total	**636**	**234**

*Source*: Sekondi-Takoradi Metropolitan Assembly Annual Report (2018)

## Procedure and measure

### Data collection instrument

*Organizational culture*: This section examines the psychological features, way of life, beliefs, values, social mindsets and habits of coworkers. The four components of organizational culture, such as consistency, employee involvement in the business, employee adaptability to corporate culture and the institution's values and mission, were used to assess this characteristic (see Table 2). The measures and results of the sub-scales are included in Appendix 2.

*Employee Productivity*: Employee productivity refers to an employee's output to the resources available to them in a given organization. This variable was also measured by the number of hours worked and the achievement of employees' job targets (See Appendix 1).

A five (5) point Likert scale was used which required respondents to indicate the extent to which they agreed with the statements posed on each scale. The scale was interpreted as follows: 1 = strongly agree, 2 = agree, neutral = 3, disagree = 4, 5 = strongly disagree. In this study, 1 and 2 indicate the strongest and moderate agreement, respectively. While 4 and 5 indicate strong and moderate disagreement, 3 is interpreted as indifference.

## Results

### Reliability statistics

The consistency of results while utilizing the same ideas or methodologies in different ways is measured by reliability [41]. The Cronbach alpha was used to assess the consistency of each variable's measures. The Cronbach alpha levels ranged from 0.97 to 0.63, with 0.97 being the highest and 0.63 being the lowest. In establishing the reliability of measures, a Cronbach alpha of 0.65 to 0.8 is appropriate [41]. Table 4 shows the results of the reliability test.

**Table 4:** Reliability test results

Variable	Cronbach alpha	No. of items
Consistency	0.63	3
Involvement	0.89	3
Adaptability	0.97	3
Mission	0.95	3
Employee productivity	0.76	7

*N* = 132

### Control variables

The background information of respondents was considered as relevant and the weight of their contribution of the total variance of the dependent variable will be lost if not controlled for. Consequently, these variables were included in the regression model. They are gender, age, education and experience. The results showed that 81 were males and 51 were females. The education variable showed that the majority had a degree (62.9%), polytechnic certificate (17.4%), master’s (12.1%) and elementary education (7.6%). The experience variable showed that 39.4 percent had worked for more than 5 years, 18.9 percent worked between 3 and 5 years, few worked for less than a year (10.6%). While 31.1 percent worked between 1 and 3 years. This implies that many of the respondents have worked for a longer period and are expected to be more accustomed to the operations and culture of the institution.

### Descriptive statistics

This section of the study presents the research findings using measures of central tendencies such as means, standard deviation, minimum and maximum values of the sub-indicators of organizational culture and employee productivity. First, the results are presented in Table 5.

**Table 5:** Descriptive statistics summary

Variable	Mean	Std Dev	Min	Max
Employee Productivity	3.0369	0.808	1.5	4
Consistency	1.641	0.657	1	3
Involvement	2.303	0.505	1	3.5
Adaptability	2.369	0.991	1	4
Mission	2.361	1.481	1	5
Total Org. culture	2.168	0.483	1.25	3.17

*Source*: *N* = 132

The results in Table 5 showed that employee productivity had a Mean = 3.0369 and SD = 0.80814 indicating an indifference in employee productivity among staff of the organization as they lack the necessary resources needed to accomplish their job tasks. The result also showed that overall, consistency Mean = 1.6414 and SD = 0.65721, since many respondents strongly agreed that organizational culture is consistent. The study also showed that there is a positive culture of involvement where the Mean = 2.1414 and SD = 0.27971. Moreover, the findings from the level of adaptability indicate that respondents agreed (Mean = 2.3687; SD = 0.99127) to be “adaptable” to the organization. Mission had a Mean = 2.3611 and SD = of 1.48103 suggesting that respondents agreed moderately to a positive organizational mission culture.

### Correlation analysis

Further, the result from the correlation in Table 6 indicates that there is a negative correlation and insignificant relationship (*β* = − 0.092) between employee productivity and adaptability in the STMA. This implies that as the rate of employees' adaptation to the institution goes up, employee productivity decreases. However, the degree of the negative relationship is low and also statistically not significant. The findings also showed a positive correlation between consistency, involvement and mission on employee productivity. The level of involvement is, however, statistically significant to employee productivity but the relationship between mission (*β* = 0.097) and consistency (*β* = 0.140) on employee productivity is not statistically significant (See Table 6).

**Table 6:** Correlation analysis summary

Variable	1	2	3	4	5
1. Consistency	1				
2. Involvement	0.526***	1			
3. Adaptability	− 0.393***	− 0.342***	1		
4. Mission	− 0.452***	− 0.491***	0.678***	1	
5. Employee prod.	0.140	0.342***	− 0.092	0.097	1

***Correlation is significant at the 0.01 level (2-tailed); *N* = 132

To further test the relationship between organizational culture as a combined variable by collapsing the sub-measures (See Table 7), the results show a positive and statistically significant relationship at *p* = 0.06 or *p* < 0.01 which was above the prediction for this study which is 0.05 or *p* < 0.05. Hence, a decision was taken to not to include this model since the regression results showed that organizational culture as a combined variable (consistency, involvement, adaptability and mission) was able to explain a total variance of 0.027 or 2 percent in the dependent variable: employee productivity, suggesting that the four indicators (involvement, consistency, adaptability and mission) were unique and better predictors in measuring organizational culture as individual variables.

**Table 7:** Correlation matrix for main variables

Variable	1	2
1. Organizational culture	1	
2. Employee prod.	0.164*	1

*Correlation is significant at the 0.01 level (2-tailed); *N* = 132

### Regression analysis

A model summary is also presented in Table 8 to ascertain the extent to which the model predicts employee productivity.

**Table 8:** Regression summary results

Model	Variable	B	Std− error	Beta	T− stat	*P*− value	Summary statistics
1	(Constant)	1.000	0.426		2.349	0.020	*R*^2^ = 0.253
	Consistency	0.008	0.116	0.006	0.067	0.947	Adj *R*^2^ = 0.230
	Involvement	0.813	0.153	0.506	5.305	0.000	SE = 0.7093
	Adaptability	− 0.230	0.086	− 0.283	− 2.683	0.008	F = 10.758
	Mission	0.295	0.062	0.541	4.768	0.000	df = 127
							*P* = 0.000
2	(Constant)	0.896	0.622		1.441	0.152	*R*^2^ = 0.288
	Consistency	0.038	0.119	0.031	0.320	0.750	Adj R^2^ = 0.222
	Involvement	0.776	0.159	0.485	4.879	0.000	SE = 0.7127
	Adaptability	− 0.228	0.087	− 0.280	− 2.610	0.010	*F* = 4.403
	Mission	0.294	0.064	0.538	4.619	0.000	df = 127
	Gender	0.129	0.135	0.078	0.955	0.342	*P* = 0.000
	Age	0.014	0.008	0.166	1.709	0.090	
	Education	− 0.034	0.187	− 0.031	− 0.182	0.856	
	Experience	− 0.102	0.073	− 0.134	− 1.396	0.165	Durbin Watson = 1.844

Dependent variable: total employee productivity

1. Predictors: (constant), mission, consistency, involvement and adaptability

2. Predictors: (constant), mission, consistency, involvement, adaptability, age, education, gender, experience

The model summary shows that with exception of consistency, all the three indicators of organizational culture, mission (*β* = 0.541, *p* = 0.000) contributed the highest in explaining employee productivity, followed by involvement (*β* = 0.506, *p* = 0.000), while adaptability negatively impacted the dependent variable showing that a unit increase in adaptability leads to the corresponding decrease of − 0.283 in employee productivity using the standardized coefficient. However, the entire model was statistically significant at *p* ≤ 0.01. Also, the collective effect of the model showed that independent variables (involvement, consistency, adaptability and mission) provided a total variance of 25.3 percent (*R*^2^ = 0.253) in the dependent variable. The individual effects of the relationship between organizational culture and employee productivity are ascertained.

In the second model, control variables were introduced and the model depicts an improvement in the total variance explained (*R*^2^ = 0.288) suggesting a unit change of 0.035 or 3.5 percent effect on the dependent variable. The F-statistics and SE demonstrate a good fit of the dataset. However, only age was statistically significant at *p* ≤ 0.1 and the remaining control variables had no significant effect on employee productivity.

From the regression results, the research hypotheses (H_1_ and H_4_) were statistically significant at *p* = 0.000 or *p* ≤ 0.01 while H_3_ was statistically significant but negative; hence, the hypothesis is retained, whereas H_2_ and H_5_ were not significant in explaining the changes in employee productivity. Although the initial prediction was at *p* ≤ 0.05, the actual results demonstrate the direction of the relationship which is positive; hence, they are supported by the initial prediction. While H_2_ and H_5_ failed to support the prediction based on the literature review, these two hypotheses suggest that the null hypotheses are true.

## Discussion

The study sought to test the effect of organizational culture on employee productivity at the STMA. The findings reveal that the mission and vision of the organization to a large extent do influence employee productivity which is consistent with previous studies [20]. The findings on the mission of organizational culture agree with the theoretical cultural model of [19] which showed that organizations that perform better are those that have a good appreciation of their organization’s goals and mission; hence, they can track their targets to increase productivity.

It is also evident from the findings that the organization engages employees and involves them in decision-making to improve organization goal attainment [18]. As a result, employees continue to remain committed and are more willing to work in the organization for a longer period demonstrating the significance of low employee attrition. The culture of involvement contributes to productivity levels as well as employees' ability to adapt to the organization’s mission and goals which also influences the productivity levels of staff. Similarly, workers' understanding and knowledge of the mission and goals of the organization is the first step toward ensuring that people are conversant with the direction of the organization which occurs in staff involvement programs during the performance year and review [23, 48, 50].

Also, the culture of the STMA is consistent with the goals of the organization because employees can share in the attributes of the institution, and they take up assignments within the scope of their abilities and skills. The consistency of culture further indicates that staff behaviors are entrenched in the values of the institution. The presence of a culture of consistency is likely to ensure a stable environment for higher productivity [27, 30].

It is also in consonance with McGregor’s Theory X and Y which demonstrates that when management can provide the needed resources and effectively organize employees by making available a stable working environment, assisting employees by giving them the right values and mission, creating the culture of involvement and adaptability, employees become committed and can achieve the corporate goal [34]. The finding is also consistent with the conceptual framework which provides that to achieve higher employee productivity, employees must be involved, adaptable and consistent in their daily work activities as suggested by [16].

Overall, the findings on organizational culture and employee productivity, revealed a positive relationship between consistency, involvement and mission on employee productivity while there is a negative relation between adaptability and employee productivity. The findings show that as employees remain consistent and involved in the decision-making process of the organization, productivity level increases [45, 49, 51]. Moreover, if employees become more adaptable to the organization, employee productivity reduces. This is possible as employees may become too comfortable in the organization and may pretend not to work. Further, when employees become conversant with the mission and values of the organization, their productivity levels also go up.

Moreover, the hypothesis testing revealed that involvement (H_1_), adaptability (H_4_) and mission (H_3_) as predictors of employee productivity are statistically significant such that they impact the productivity of employees positively. However, consistency (H_2_) is not statistically significant in determining employee productivity. Overall, three of the indicators of organizational culture do affect the productivity of employees of the STMA. This conforms with the works of [5, 8, 52] who found a similar statistically significant relationship between organizational culture and productivity in the National Agency for Food and Drugs Administration and Control in Nigeria. A similar study by [3] conducted in the telecom sector of Pakistan supports the findings of the work as organizational culture was found to be statistically significant on employee productivity. The finding also agrees with the work of [42] who found a significant positive relationship between involvement, adaptability and mission on employee productivity among information technology companies in Romania.

## Conclusion

The study looked into the impact of organizational culture on employee productivity among STMA employees. Based on the findings, it can be concluded that effective organizational culture plays an important role in employee productivity. It is worth noting that some aspects of Denison’s model have explanatory power on employee productivity. For instance, employees in the organization demonstrate their involvement in decision-making which enables them to create their sense of identity while they internalize the mission of the STMA which contributed more to their productivity levels. As a result, they are comfortable working in the organization for a longer period. But when employees feel comfortable working in an institution for a longer time, it is negatively related to their productivity levels. This relationship, however, does not suggest that adaptability may not support productivity at the organizational level. Notwithstanding, the experiences of employees on the job were seen in the way they translated the mission and goals of the Assembly to higher levels of productivity.

The findings of this study point to two key implications. A major focus of policymakers in Ghana has been to address low productivity in the public sector with the implementation of wide reforms resulting in only minimal gains. With this study’s finding showing that organizational culture of mission, involvement and consistency are central to employee performance, policymakers need to develop strong organizational culture practices in local governments such as compliance and a reward-based system geared toward building resilience in culture dynamics as a tool to foster productivity of employees. Local authorities need to design their mission to link with the organizational culture to ensure that employees can adapt to task performance. The ability to adapt to an organization, if well integrated, will enhance employee productivity.

Secondly, the policy implementers and top management should improve their organizational culture by allowing both horizontal and vertical participation of employees in decision-making since the culture of involvement has a strong effect on employee productivity. In this respect, organizations should involve employees in decision-making to create a sense of belonging as employees stay longer with the organization. There is the need to push for increased ownership of the mission by employees through their active participation in setting key performance targets during the performance cycle to build a strong culture.

Despite the robust nature of the findings, the study has some limitations common with the use of survey designs in collecting primary data. Firstly, the study’s use of a survey design with a structured instrument may be biased in measuring some of aspect of the Ghanaian culture. Secondly, the measures adapted from Denison’s model on culture of adaptability may be skewed since the study found that the more employees are adapted to their organizations, the more likely their productivity levels are negatively affected. These findings should be interpreted with caution since additional interviews would have added more information on why this is the case. In this regard, the study recommends for future studies to focus on mixed-methods research design to mitigate this shortfall. Thirdly, the single case used in this study is small considering that there are about six (6) Metropolitan Assemblies in Ghana, a comparative study would have made the findings more interesting. Future studies can employ a comparative approach to look at the remaining Metropolitan Assemblies.

## Data Availability

The datasets used and/or analyzed during the current study are not publicly available due to the privacy of the participants who provided the information for the study but are available from the corresponding author on reasonable request.

## Appendix 1: employee productivity scale

To what extent do you agree to the following as the culture in Sekondi-Takoradi Metropolitan Assembly?

Strongly Agree (SA) -1; Agree (A)-2; Neutral (N)-3; Disagree (D)-4; Strongly Disagree (SD)-5.

## Appendix 2: organizational culture scale

To what extent do you agree to the following as the culture in Sekondi-Takoradi Metropolitan Assembly?

Strongly Agree (SA)-1; Agree (A)-2; Neutral (N)-3; Disagree (D)-4; Strongly Disagree (SD)-5.

## Abbreviations

ANOVA: Analysis of variance
COVID-19: Coronavirus 2019
OLS: Ordinary least squares
NAFDAC: National Agency for Food and Drugs Administration and Control
SPSS: Statistical package for social scientists
STMA: Sekondi-Takoradi Metropolitan Assembly
